# Brain‐derived neurotrophic factor drives muscle adaptation similar to aerobic training in mice

**DOI:** 10.1096/fj.202402421R

**Published:** 2025-01-24

**Authors:** Alexander D. Brown, Alexander D. Marko, Daniel M. Marko, Bradley J. Baranowski, Sebastian Silvera, Michael S. Finch, Alex J. Yang, Roopan Dhaliwal, Chantal R. Ryan, Brian D. Roy, Val A. Fajardo, Rebecca E. K. MacPherson

**Affiliations:** ^1^ Department of Health Sciences, Faculty of Applied Health Sciences Brock University St. Catharines Ontario Canada; ^2^ Department of Kinesiology Faculty of Applied Health Sciences, Brock University St. Catharines Ontario Canada; ^3^ Centre for Neuroscience Brock University St. Catharines Ontario Canada

**Keywords:** aerobic exercise, brain‐derived neurotrophic factor, contraction, mitochondria, skeletal muscle

## Abstract

This study, in vivo and in vitro, investigated the role of brain‐derived neurotrophic factor (BDNF) in skeletal muscle adaptations to aerobic exercise. BDNF is a contraction‐induced protein that may play a role in muscle adaptations to aerobic exercise. BDNF is involved in muscle repair, increased fat oxidation, and mitochondrial biogenesis, all of which are adaptations observed with aerobic training. The purpose of this study was two‐pronged and investigated the skeletal muscle BDNF response to (1) acute and (2) chronic exercise in male C57BL/6J mice. It also examined if chronic BDNF treatment resulted in similar adaptations to chronic exercise. In aim 1, mice underwent a 2 hr. treadmill exercise bout. In aim 2, mice were assigned to one of four groups: (1) control (CON); (2) endurance training (ET; treadmill running 1 h/day, 5 days/wk); (3) BDNF (BDNF; 0.5 mg/kg·bw, 5 days/wk); (4) endurance training and BDNF (ET + BDNF) for 8 weeks. Results demonstrated that the soleus (SOL) had higher BDNF content compared with the extensor digitorum longus (EDL) and that SOL BDNF increased with acute exercise. After chronic exercise and BDNF treatment, treadmill testing to exhaustion demonstrated a main effect of BDNF and ET on increasing exercise capacity. In vitro contractile assessment of the EDL revealed BDNF treatment resulted in similar increases in the max rate of relaxation as ET. EDL force‐frequency analysis showed ET + BDNF produced higher force than CON and BDNF, indicating an additive effect. BDNF increased EDL mitochondrial proteins, COXIV, and CS. These results demonstrate that BDNF contributes to muscle adaptations observed with ET.

## Graphical abstract

This study investigated BDNF's role in skeletal muscle adaptations to aerobic exercise in mice. It examined acute and chronic exercise effects on BDNF response and compared chronic BDNF treatment to exercise adaptations. Results showed increased BDNF in soleus after acute exercise, improved exercise capacity with chronic exercise and BDNF treatment, enhanced muscle relaxation and force production, and increased mitochondrial proteins. The study concludes that BDNF contributes significantly to muscle adaptations observed in endurance training.

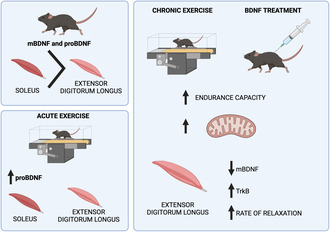

## 1 INTRODUCTION

Brain‐derived neurotrophic factor (BDNF) has been well characterized in the central nervous system for playing a role in synaptic plasticity, neuronal differentiation, maturation, proliferation, and survival.^1^ BDNF, first synthesized as pro‐BDNF (~32 kDa) and then further cleaved to a mature mBDNF form (~14 kDa), is also expressed in peripheral tissues such as skeletal muscle.^2^, ^3^, ^4^, ^5^, ^6^, ^7^, ^8^, ^9^ However, the roles that BDNF has in skeletal muscle are diverse with reports indicating that BDNF plays a role in satellite cell differentiation and muscle repair,^5^, ^10^ AMPK activation and fat oxidation,^2^, ^11^ mitochondrial biogenesis,^3^, ^11^, ^12^ as well as regulating fiber‐type composition.^13^, ^14^, ^15^, ^16^, ^17^ BDNF is an exercise‐induced protein that increases in the periphery with acute and chronic exercise,^18^, ^19^, ^20^, ^21^, ^22^, ^23^ and its gene and protein content increase in skeletal muscle with contraction, and with acute and chronic exercise.^2^, ^17^, ^23^, ^24^, ^25^, ^26^, ^27^, ^28^ Given the potential roles of BDNF in skeletal muscle physiology, it is possible that exercise‐induced BDNF plays a role in the beneficial muscle adaptations that accompany exercise training.

The response of skeletal muscle to endurance training (ET) is well documented and involves enhancements in the capacity and efficiency for energy production (enhanced fatty acid oxidation and mitochondrial biogenesis) and fatigue resistance with a shift toward a more oxidative fiber‐type^29^, ^30^—all adaptations that are similar to the skeletal muscle response to BDNF. Matthews et al.^2^ demonstrated that BDNF mRNA and protein expression were higher in human skeletal muscle after exercise and further demonstrated that BDNF mRNA and protein expression were higher in culture C2C12 muscle cells that were electrically stimulated. It was further demonstrated that BDNF activates AMPK signaling and enhances fatty acid oxidation in vitro in L6 cells and ex vivo in intact muscle (rat extensor digitorum longus—EDL).^2^ These findings have been confirmed in C2C12 myotubes where BDNF treatment promoted fatty acid oxidation in a dose‐dependent manner as well as in muscle‐specific BDNF knockout mice where fatty acid oxidation was reduced.^11^ These BDNF‐induced increases in fat oxidation are likely due to acute changes related to AMPK signaling (ACC phosphorylation^2^) and to chronic adaptations at the mitochondria that are similar to what is observed with acute and chronic exercise. In support of this hypothesis, BDNF also regulates the expression of peroxisome proliferator‐activated receptor γ coactivator 1α (PGC‐1α), a key regulator of mitochondrial biogenesis and a recognized target for developing exercise‐mimicking drugs.^11^, ^31^, ^32^ BDNF‐treated myotubes display a higher content of several mitochondrial proteins (cytochrome c, succinate dehydrogenase, and pyruvate dehydrogenase) and mitochondrial DNA. Further oxygen consumption rate, basal and maximal mitochondrial respiration, and ATP production are all elevated in BDNF‐stimulated cells.^11^ In terms of in vivo evidence linking exercise, BDNF, and mitochondrial adaptations, recent work in mice reported higher BDNF and phosphorylated TrkB in the soleus (SOL) muscle after 8 weeks of moderate‐intensity running. These results were accompanied by higher phosphorylated AMPK and PGC‐1α content.^31^ Finally, the authors demonstrated that exercise training resulted in a longer distance run in a treadmill test to exhaustion.^31^ Collectively, the current state of the literature suggests that BDNF may mediate some of the adaptive changes that occur in skeletal muscle after regular aerobic exercise. However, to our knowledge, this possibility has never been thoroughly examined directly.

The purpose of this study was to investigate the role of BDNF in skeletal muscle adaptations resulting from aerobic exercise. To accomplish this purpose, the study was divided into two aims. Aim 1 characterized the acute effects of treadmill exercise on BDNF content in oxidative (SOL) and glycolytic (EDL) skeletal muscles and Aim 2 compared the chronic effects of BDNF treatment with those of endurance training (ET) on muscle adaptations. It was hypothesized that acute treadmill exercise would increase BDNF content in both muscle types, particularly in the oxidative muscle. It was further hypothesized that chronic BDNF treatment would replicate the skeletal muscle adaptations observed with ET, suggesting a causal role for BDNF in exercise‐induced muscle plasticity.

## 2 MATERIALS AND METHODS

### 2.1 Animals

All experimental procedures were approved by the Brock University Animal Care Committee and followed the guidelines of the Canadian Council on Animal Care. Male 10‐week‐old C57BL6/J mice were purchased from The Jackson Laboratory (Bar Harbor, Maine, USA). Mice were housed in groups of four and acclimatized for 7 days in the Brock University Comparative Biosciences Facility. All mice were kept on a 12‐h light: 12‐h dark cycle and had ad libitum access to food and water through the entirety of the study.

### 2.2 Acute exercise protocol and tissue collection

Mice were acclimated to a motorized treadmill running for 10 min at 15 m/min at a 5% incline on two successive days (Columbus Instruments, EXER‐6M Treadmill). After 48 h of acclimatization, mice ran for 2 h at 15 m/min at a 5% incline during the light cycle.^33^, ^34^, ^35^, ^36^, ^37^ To examine the acute exercise response of BDNF, the SOL and EDL were collected 15 min post‐ the 2‐h treadmill running bout (*n* = 7). Sedentary non‐exercise control mice were also euthanized and prepared for muscle collection concurrently (*n* = 6). Mice were anesthetized with an intraperitoneal injection of sodium pentobarbital (6 mg/100 g body weight). Blood was collected via cardiac puncture and the mice were euthanized via exsanguination.

### 2.3 Chronic Exercise and BDNF Protocol

Male C57BL6/J mice were assigned into one of four groups (*n* = 12 per group): (1) control (CON); (2) endurance training (ET); (3) BDNF; or (4) endurance training and BDNF (ET+BDNF). The study interventions took place over 8 weeks and body mass (g) and food intake (g) were recorded weekly. Prior to the beginning of the study, there were no differences in body mass between the groups (CON: 24.6 ± 0.44 g; ET: 24.1 ± 0.83 g; BDNF 24.4 ± 0.51 g; ET+BDNF: 24.0 ± 0.51 g; *p* > .05).

Mice within the control group remained sedentary throughout the 8‐week intervention. However, to reduce the effects of increased handling and movement to and from the treadmill that the other experimental groups experienced these mice were handled 5 days/week and their cages brought to the treadmill room. Mice within the exercise group underwent endurance exercise training 5 days/week for 8 weeks. Mice were acclimated to treadmill running during two short low‐intensity sessions (10 min, 15 m/min, 5% incline). Training commenced 48 h after the last acclimation session. Training consisted of treadmill running for 1 h per day, 5 days per week for 8 weeks (trained during the light cycle, between 09:00 and 11:00 a.m.). Training was progressive and increased from 20 m/min at a 10% incline in week 1–25 m/min at a 20% incline in week 8.^38^, ^39^, ^40^ Mice in the BDNF group underwent rotating subcutaneous injections of BDNF (0.5 mg/kg body mass) 5 days per week for 8 weeks. This dose of BDNF has previously been shown to result in similar serum BDNF concentrations as endurance training.^38^ Mice in the combined ET + BDNF group underwent treadmill training as described for the ET group as well as BDNF administration as described in the BDNF group. The control and ET groups received an equivalent volume of saline via subcutaneous injections (10 mM phosphate and 150 mM NaCl, pH 7.0).^38^

### 2.4 Treadmill fatigue testing

The treadmill exhaustion test was adapted from the protocol outlined by Castro and Kuang.^41^ Briefly, the treadmill was set to an incline of 10%. The mice were placed in the appropriate lane and allowed to explore the environment with the treadmill off. A warmup was conducted at a speed of 12 m/min for a duration of 5 min; following this period, the test was initiated. Treadmill speed was set to 12 m/min and then further increased by 2 m/min every 2 min until the maximum speed of 46 m/min was reached. The test was conducted until the mice were exhausted. In the current study, exhaustion was defined as the inability of the animal to run on the treadmill for 10 s consecutively despite slight mechanical prodding. The speed and time at which exhaustion occurred was recorded. Distance, work, and power were also determined. Work and power were calculated as:

$$\begin{array}{c} \text{Work}\left(J\right)=\text{body mass}\;\left(\mathrm{kg}\right)\times \text{gravity}\;\left(9.81\;m/\text{sec2}\right)\\ \;\times \text{vertical speed}\;\left(m/\sec\times \text{angle}\right)\times \text{time}\;\left(\sec\right). \end{array}$$

$$\text{Power}\left(W\right)=\text{work}\;\left(J\right)/\text{time}\;\left(\sec\right).$$

### 2.5 Glucose tolerance test

All mice were fasted 6 h before testing, with ad libitum access to water. Mice were injected with a weight‐adjusted intraperitoneal bolus injection of glucose (2 g/kg body mass), and changes in blood glucose were monitored from tail blood using a handheld glucometer (FreeStyle Lite, Abbott Laboratories). Before injection, baseline blood glucose concentrations were determined (Time 0). Blood was tested at 15‐, 30‐, 45‐, 60‐, 90‐, and 120‐min post glucose injection.

### 2.6 Tissue collection

Mice were anesthetized with an intraperitoneal injection of sodium pentobarbital (6 mg/100 g body weight). Blood was collected via cardiac puncture and the mice were euthanized via exsanguination. The SOL and EDL from both the left and right side were collected for contractility and fiber typing, as well as snap frozen for biochemical analysis.

### 2.7 In vitro muscle contractility

Intact SOL and EDL muscles were carefully dissected and suspended in a bath with oxygenated Tyrode solution (95% O_2_, 5% CO_2_) containing 121 mmol/L NaCl_2_, 5 mmol/L KCl, 24 mmol/L NaHCO_3_, 1.8 mmol/L CaCl_2_, 0.4 mmol/L NaH_2_PO_4_, 5.5 mmol/L glucose, 0.1 mmol/L EDTA and 0.5 mmol/L MgCl_2_, pH 7.3 and maintained at 25°C. Force frequency curve (FFC) experiments were done, specifically measuring maximal isometric force across the range of stimulation frequencies. For the EDL; 1, 20, 60, 100, and 150 Hz were used. Each stimulation was 400 ms in length, with a rest period of 60 s between each contraction. And 5 min after the last stimulation at 150 Hz, a fatigue protocol of 70 Hz volleys every 2 s for 5 min was conducted. FFC for the SOL was conducted with 1, 5, 10, 20, 30, 40, 60, 80, and 100 Hz. For FFC analysis, peak isometric force was measured and then normalized to the calculated cross‐sectional area after measuring muscle length and weight as previously described.^42^ We also measured the maximal rates of force development (+dF/dt) and relaxation (−dF/dt) were also determined during a twitch and tetanic contractions. For fatigue analysis, we normalized the data to the initial 70 Hz contraction, which served as our initial or baseline strength. The area‐under‐the fatigue curve was measured to determine the amount of force produced over the 5 min protocol. All contractile experiments were conducted at a muscle length of *L*_o_.

### 2.8 Western blotting

Samples were homogenized (FastPrep, MP Biomedicals, Santa Ana, CA) in 20 volumes of NP40 Cell Lysis Buffer (Life Technologies; CAT# FNN0021) supplemented with 34 μL phenylmethylsulfonyl fluoride and 50 μL protease inhibitor cocktail (Sigma; CAT# 7626‐5G, CAT# P274‐1BIL). Homogenized samples were then centrifuged at 4°C for 15 min at 12 000 *g*, after which the supernatant was collected, and protein concentration was determined using a Bicinchoninic acid assay (Sigma‐Aldrich—B9643, VWR—BDH9312). The samples were prepared to contain equal concentrations (1 μg/μL) of protein in 2× Laemmli buffer and placed in a dry bath at 100°C for 5 min. Samples (10–20 μg of protein) were loaded and separated on 10% SDS‐PAGE gels for 90 min at 120 V. Proteins were then wet‐transferred onto a nitrocellulose membrane at 100 V for 60 min. Membranes were blocked with 5% non‐fat powdered milk in Tris‐buffered saline/0.1% Tween 20 (TBST) for 1 h at room temperature. The appropriate primary antibody (1:1000 ratio) was then applied and left to incubate overnight on a shaker at 4°C. Following primary incubation, the membranes were then washed with TBST 3 × 5 min and incubated with the corresponding secondary antibody conjugated with horseradish peroxidase (Jackson ImmunoResearch, 1:2000 ratio) for 1 h at room temperature. Signals were detected using enhanced chemiluminesence and were subsequently quantified by densitometry using a FluorChem HD imaging system (Alpha Innotech, Santa Clara, CA). A representative Ponceau S stain was measured and analyzed for each membrane to ensure equal loading (<10% variability across the membrane).^43^ The proteins that were examined included BDNF (1:1000, ABCAM cat. no. 10819), TrkB (total (1:1000, Cell Signaling cat. no. 4603) and phosphorylated TrkB (1:1000, Abcam cat. no. ab109684)), mitochondrial proteins; citrate synthase (CS, ab96600, 1:1000 dilution; Abcam), COXIV (Cytochrome C oxidase subunit 4; ab16056, 1:1000 dilution; Abcam) and PDH (AB52082, 1:1000 dilution; Millipore), and PGC‐1alpha (AB3242, 1:1000 dilution; Millipore).

### 2.9 Histochemical and immunofluorescence analysis

Each OCT‐embedded muscle sample was mounted on a chuck using OCT and clamped in a ThermoFischer Scientific Cryostat Microtome, maintained at −25°C. The sample was positioned at 90° to the cutting blade. Cross sections of the muscle belly were sliced at 40 μm thickness and transferred to a vectabonded slide. And four cross sections per sample were transferred to each slide and incubated at 37°C for 30 min. Slides were then stored at −80°C until analysis.

Frozen slides were removed, defrosted, and placed on a wet paper towel to air dry at RT. Muscle sections were outlined with a pap pen. Sections were incubated for 1 h in 100 μL blocking solution (10% goat serum in PBS) and carefully blotted dry using kimwipes. Primary antibody cocktail against MHCI at 1:50 (BA‐F8), MHCIIa at 1:600 (SC‐71), and MHCIIb at 1:100 (BF‐F3) diluted in 10% blocking solution was formulated. Slides were incubated in a primary antibody cocktail overnight in a dark container. Slides were washed in PBS 3 × 5 min in Columbia jar on a shaker at 150 rpm. Slides were then blotted dry. The secondary antibody cocktail was formulated, containing Alexa Fluor 350 IgG_2b_ at 1:500 (blue‐MHCI); Alexa Fluor 488 IgG_1_ at 1:500 (green‐ MHCIIa); Alexa Fluor 555 IgM at 1:500 (red‐MHCIIb). Muscle sections were incubated in 100 mL of secondary antibody cocktail at room temperature in a dark room for 1 h. Slides were washed in PBS 3 × 5 min in Columbia jar and blotted dry. 15 mL of Prolong® Gold antifade Reagent was applied to the middle of the muscle sections and a coverslip was applied. Nail polish was used to secure the corners of the coverslip and left to dry for 10 min.

Muscle sections were visualized using Cytation™ 5 Biotek Imager equipped with Red (Excitation: BP 545/25 nm; Emission BP 605/70 nm), Green (Excitation: BP 470/40 nm; Emission BP 525/50 nm), and Blue (Excitation: BP365/12 nm; Emission LP 397 nm) filters. Images were captured at 40x magnification. Images were analyzed using ImageJ for fiber percentage distribution, number, and CSA. These methods were adapted from Bloemberg and Quadrilatero (2012)^44^.

### 2.10 Statistical analysis

The response to acute exercise was analyzed via a student *t*‐test. For the chronic exercise study, body mass and glucose tolerance test results were recorded, and a two‐way repeated measures (RM) ANOVA analysis was performed. For the glucose tolerance tests, two‐way RM ANOVA was followed by calculating the area under the curve and conducting a two‐way ANOVA based on ET by BDNF treatment. Differences between total and phosphorylated protein content as well as differences in muscle contractility were analyzed using two‐way ANOVAs. Significant interactions were followed up with Tukey post hoc analysis. In cases where data were not normally distributed, data were logarithmically transformed. Data are expressed as means ± SEM with significance set at *p* < .05.

## 3 RESULTS

### 3.1 Soleus BDNF content is higher compared with the EDL and is responsive to acute exercise

**FIGURE 1:**
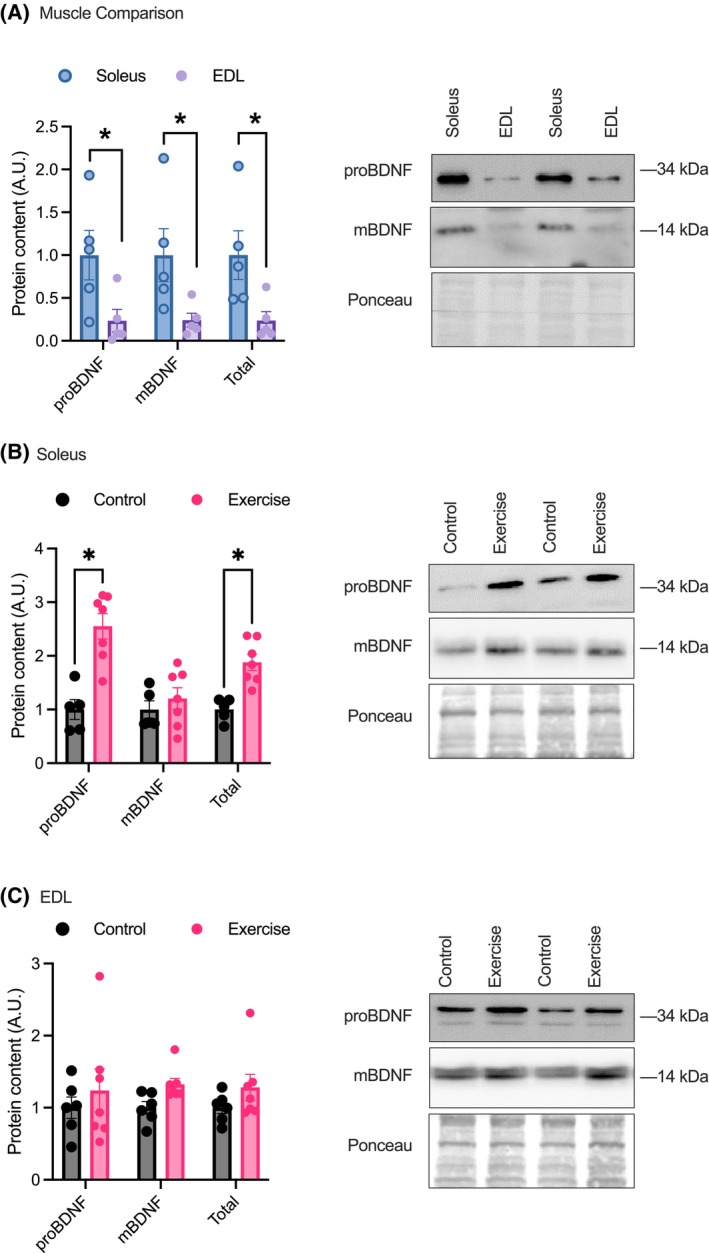
Soleus and EDL muscle BDNF content at rest and in response to acute exercise. (A) Soleus and EDL pro‐ and mature BDNF content. Soleus *n* = 5 and EDL *n* = 5. Acute exercise response of BDNF in the soleus (B) and EDL (C). Control *n* = 6 and exercise *n* = 7. Representative western blots are shown below each figure. *p < .05 significance was determined using a two‐tailed *t*‐test. All data are presented as a mean ± SEM.

The SOL was found to have higher proBDNF and mBDNF protein content when compared with the EDL (*p* < .05; Figure 1A) in sedentary mice. To examine the acute exercise response of BDNF, the SOL and EDL were collected 15 min after a 2‐h treadmill running bout. The SOL of exercised animals had a higher content of proBDNF compared with sedentary SOL muscle (*p* < .05; Figure 1B). There was no difference in SOL mBDNF protein content between groups. The EDL muscle showed no difference in proBDNF or mBDNF levels when comparing exercised EDL to sedentary EDL (Figure 1C).

### 3.2 Effect of endurance training and BDNF treatment in SOL and EDL

**FIGURE 2:**
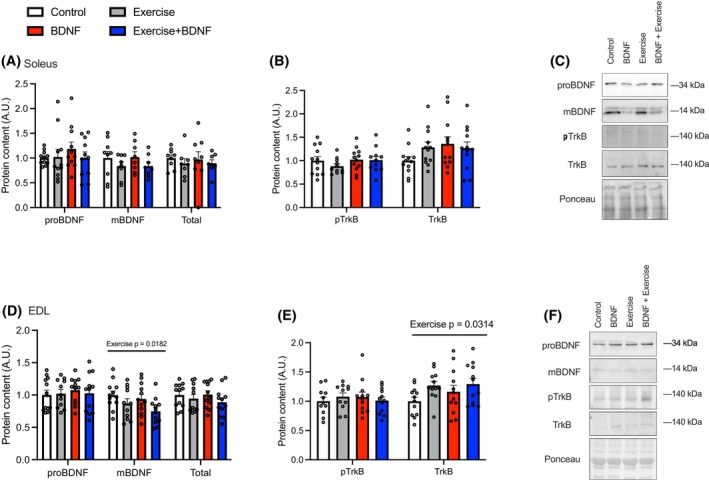
BDNF content and signaling post‐endurance training and BDNF treatment. (A) Soleus pro‐ and mature BDNF content. (B) Phosphorylated and total TrkB content. (C) Representative western blot images. (D) Soleus pro‐ and mature BDNF content. (E) Phosphorylated and total TrkB content. (F) Representative western blot images. The main effects are shown above graphs. *n* = 8–12/group. Values represented as mean ± SEM.

In the SOL, no differences were observed in BDNF (Figure 2A) or total and phosphorylated TrkB (Figure 2B) protein content across CON, BDNF, ET, and BDNF+ET groups. In the EDL, it was found that ET resulted in lower mBDNF content (Figure 2D; *p* = .0182, main effect). There was also a main effect of ET for increasing TrkB (*p* = .0314; Figure 2E) protein content.

### 3.3 Treadmill training and BDNF treatment alter body mass, food intake, and treadmill time to exhaustion

**FIGURE 3:**
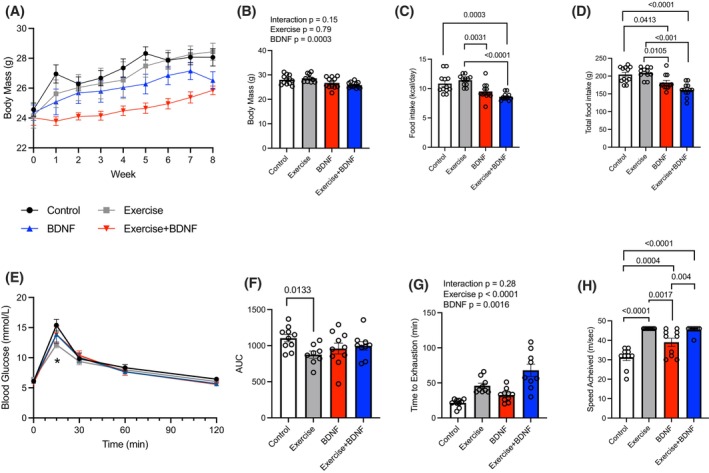
Whole body characterization of BDNF treatment in comparison to endurance training. (A) Weekly body mass. (B) Terminal body mass. (C) Average food intake. (D) Total food intake. (E) Glucose tolerance test. (F) glucose tolerance test represented as area under the curve. (G) time to exhaustion. (H) speed achieved. *n* = 8–12/group. Values are presented as mean ± SEM. Significance is indicated in figures.

Differences in body mass between groups were evident in week 1 of the interventions where the control group was heavier than the combination group (Figure 3A; *p* < .05). The CON group remained heavier during weeks 2, 3, and 4 (*p* < .005), week 5 and 6 (*p* < .0001), and week 7 and 8 (*p* < .005 and *p* < .05). The influence of BDNF on body mass is further observed within the exercise groups, where the ET+BDNF group had a lower body mass compared with the ET group in weeks 5 (*p* < .05), 6 (*p* < .01), 7 (*p* < .005), and 8 (*p* < .01). Final body mass was lower in the BDNF and ET+BDNF groups, indicating that BDNF plays a role in body mass regulation regardless of physical activity level (BDNF main effect *p* < .001; Figure 3B).

Average food intake (kcal/day) throughout the study was lower in the BDNF and ET + BDNF groups compared with CON and ET (Figure 3C). This was similar to total food intake for the 8 weeks where the BDNF and ET+BDNF groups consumed less food, potentially accounting for the body mass difference between groups (Figure 3D).

Glucose tolerance testing (Figure 3E) showed a lower plasma glucose at the 15‐min time point for exercise group compared with the control group (*p* < .05), demonstrating the beneficial effects of exercise training on glucose tolerance. No effect of BDNF treatment was observed. Area under the curve (AUC) analysis highlighted a significant interaction and multiple comparisons demonstrated that the ET group had a lower AUC compared with CON (*p* = .0133, Figure 3E).

Both BDNF and ET resulted in a longer time to exhaustion on the exhaustion treadmill test (main effect of BDNF *p* = .0016 and main effect of endurance training *p* < .001; Figure 3G). Analysis of the final speed achieved revealed a significant interaction where the BDNF, ET, ET+BDNF groups achieved a faster speed compared with the CON group, and the ET and ET+BDNF groups achieved a faster speed compared with the BDNF group (Figure 3H). These results demonstrate that BDNF alone can improve exercise performance on this test to exhaustion however not to the same extent as exercise.

### 3.4 Endurance training and BDNF alter muscle contractility profiles

**FIGURE 4:**
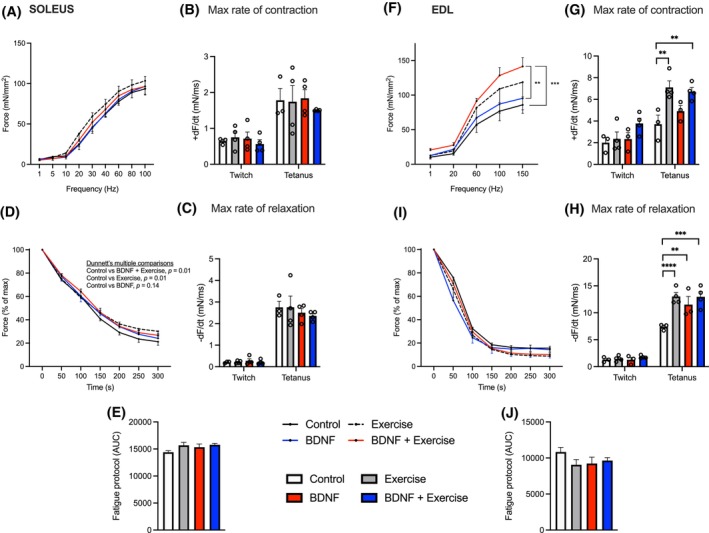
Soleus and EDL contractile properties. (A) Soleus force‐frequency curve. (B) Soleus max rate of contraction. (C) Soleus max rate of relaxation. (D) Soleus muscle fatigue curve. (E) Soleus 50% rundown. (F) EDL force‐frequency curve. (G) EDL max rate of contraction. (H) EDL max rate of relaxation. (I) EDL muscle fatigue curve. (J) EDL 50% rundown. Values represented as mean ± SEM, *n* = 4. Significance indicated by: (F) **p < .01 control versus combination; ***p < .005 control versus BDNF. (G) *p < .05 control versus BDNF.

Following the differences seen in the treadmill time to exhaustion, we examined the EDL and SOL muscles in vitro for differences in contraction and fatigue. No differences were observed in the SOL FFC, maximal rate of contraction or relaxation, or the fatigue protocol (Figure 4A–E). The EDL FFC showed a main effect for frequency (*p* < .0001) and treatment (*p* < .0005). EDL multiple comparison analysis showed that the ET+BDNF group produced more force compared with control at 100 Hz (*p* < .05) and 150 Hz (*p* < .01; Figure 4F). This was also true at 150 Hz for the combination group in relation to the force produced by the BDNF group (*p* < .05). Two‐way ANOVA analysis of EDL tetanic maximal rate of contraction (+dF/dt) and relaxation (−dF/dt) showed a main effect for ET, *p* = .001 and *p* = .004 respectively (Figure 4G,I). No differences between groups were observed in the fatigue protocol (Figure 4H,J).

### 3.5 Endurance training and BDNF influence muscle fiber‐type and cross‐sectional area

**FIGURE 5:**
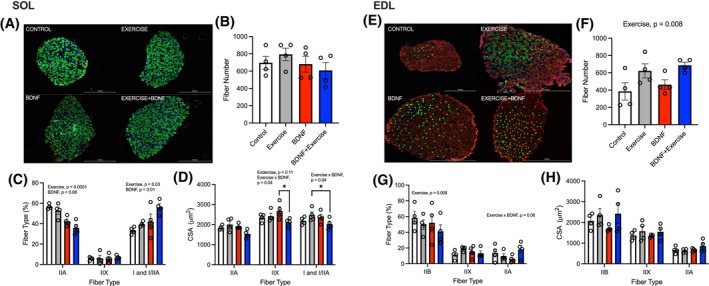
Soleus and EDL muscle fiber analysis. (A) Representative histological image of the soleus (SOL). (B) Soleus total fiber number. (C) Soleus fiber‐type percentage. (D) Soleus fiber‐type cross‐sectional area. (E) Extensor digitorum longus representative histological image (EDL). (F) EDL total fiber number. (G) EDL fiber‐type percentage. (H) EDL fiber‐type cross‐sectional area. Values represented as mean ± SEM. *p < .05. *n* = 4.

In the SOL, there were no differences between groups for fiber number (Figure 5B). There was a main effect for ET (*p* < .0001) and BDNF (*p* = .08) on type IIA and I and I/IIA percentages (Figure 5C). Type IIX, I, and I/IIA showed interactions between groups for CSA (Figure 5D). The BDNF group had a larger CSA than the ET+BDNF group in type IIX fibers (*p* = .03), and ET+BDNF group had a smaller fiber CSA than the ET groups in type I and I/IIA fibers (*p* = .04).

In the EDL, there was a main effect of ET on elevating fiber number (*p* = .008; Figure 5F) and on lowering type IIB fiber percentage (Figure 5G; *p* = .008). There was no effect of BDNF on fiber number or percent fiber‐type. However, there was an interaction that was approaching significance for type IIA fibers (*p* = .06). No other significance for fiber analysis was shown for EDL.

### 3.6 Endurance training and BDNF both increase mitochondrial proteins

**FIGURE 6:**
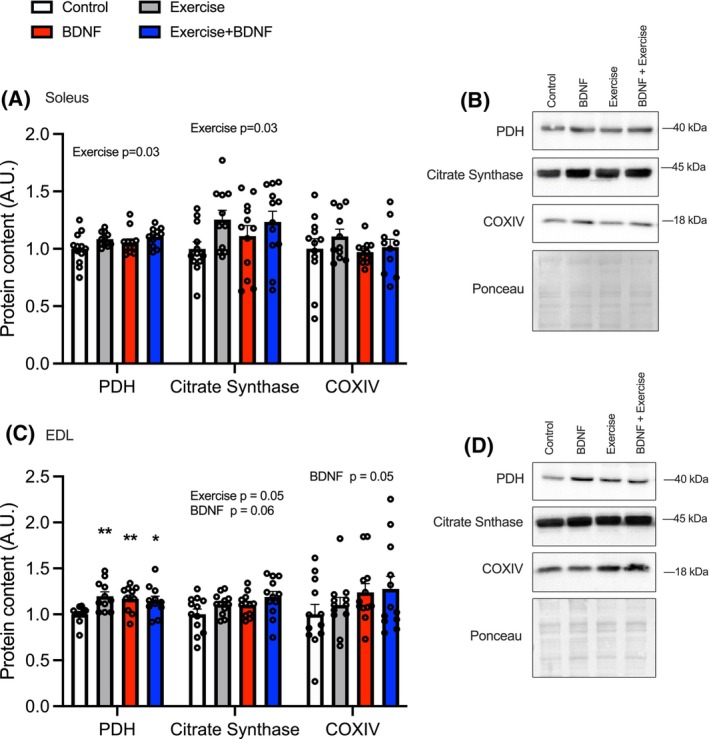
Mitochondrial protein content. (A, B) Soleus PDH, citrate synthase, and COXIV content. (C) Representative western blot images. (D, E) EDL BDNF and TrkB content. (F) Representative western blot images. Values represented as mean ± SEM. *n* = 12.

In the SOL there was a main effect for ET where the exercised groups had higher PDH (Figure 6A; *p* = .0487) and citrate synthase (*p* = .064). No effect of BDNF was observed in the SOL. In the EDL, all treatment groups had a higher PDH content compared with CON (Figure 6B). There was a main effect for ET (*p* = .05) and an approaching significant main effect of BDNF for citrate synthase content (*p* = .06). There was a main effect for BDNF on COXIV (*p* = .05).

## 4 DISCUSSION

The data presented provides a novel comparison of BDNF content and the response to acute and chronic aerobic exercise in oxidative (SOL) and glycolytic (EDL) muscle. We show that in male C57BL6/J mice, the more oxidative SOL muscle expresses more pro and mature BDNF when compared with the glycolytic EDL muscle and that acute exercise elevated proBDNF content in the SOL and not the EDL. In addition to characterizing muscle‐specific BDNF, this study provides a novel examination comparing the effects of BDNF administration to progressive ET. Overall, we demonstrate that BDNF treatment results in similar whole body (enhanced endurance performance) and muscle‐specific adaptations (muscle contractility, increased mitochondrial content) as ET. Based on these data, it is likely that peripheral and/or intramuscular BDNF is an active driver of exercise‐induced skeletal muscle adaptations.

Currently, there is conflicting evidence as to which fiber‐type has a higher BDNF expression. Given the role that BDNF plays in regulating fat oxidation and mitochondria, it would be expected that BDNF would be more highly expressed in oxidative fiber types. Previous work has demonstrated that BDNF mRNA and protein expression are higher in oxidative compared with glycolytic rodent muscle.^15^, ^16^ Work in human skeletal muscle further supports this fiber‐type distribution where pro‐BDNF protein and mRNA are expressed primarily in type I muscle fibers.^9^ However, there are also discrepant findings, with other studies indicating that BDNF is higher in glycolytic fibers. In human skeletal muscle, it was previously found that fast‐twitch glycolytic fibers (MHC IIa and IIx) expressed higher levels of BDNF when compared with oxidative fibers (MHC I).^45^ This finding was also observed in mice (B6/SJL), where the SOL had lower levels of mBDNF when compared with the plantaris,^46^ and in Wistar rats where BDNF immunostaining was stronger in type II versus type I muscle fibers.^47^ It has further been demonstrated that BDNF knockout induces a shift toward a more oxidative slow fiber type and that overexpression of BDNF results in a glycolytic fiber‐type shift.^13^ However, fiber‐type BDNF content was not measured in wild‐type mice in this study. These divergent results related to muscle−/fiber‐type‐specific BDNF expression may be due to the lack of protein‐specific reports that do not distinguish from pro‐BDNF or mBDNF. As there are functional differences between pro‐BDNF and mBDNF where pro‐BDNF binds to the p75 neurotrophin receptor and can activate apoptotic signaling while mBDNF binds to tyrosine receptor kinase B (TrkB) and can activate pathways related to growth, the identification of both isoforms in muscle should be examined.

Here, our findings again suggest that BDNF expression is fiber‐type specific, with the oxidative SOL muscle expressing higher pro‐BDNF and mBDNF content compared with the EDL. Furthermore, in response to acute exercise, proBDNF, but not mBDNF was elevated in the SOL; however, this was not maintained over time with ET. There were no changes in pro‐BDNF or mBDNF in the EDL in response to acute exercise; however, mBDNF was lowered after 8 weeks of treadmill training. Thus, our results show the muscle and fiber‐type‐specific expression patterns of pro‐ and mBDNF in a basal state and in response to acute and chronic exercise. This is important information as previous work^2^, ^13^ has not provided information related to pro and mature BDNF, likely resulting in conflicting reports of fiber/muscle‐type distribution of BDNF. Additionally, exercise training did result in a higher content of the BDNF receptor, TrkB, in the EDL‐ potentially indicating a higher sensitivity to BDNF in this muscle following ET.

It is well established that exercise training results in adaptations in muscle that contribute to the resistance of muscular fatigue,^48^ thus it is not surprising that 8 weeks of treadmill training resulted in a longer time to exhaustion in our study. Previous research in humans has examined the correlation between elevated plasma BDNF levels in trained athletes versus untrained individuals, suggesting BDNF contributes to increased physical performance markers such as VO_2max_ and power‐generating capabilities.^49^, ^50^ Our work is the first to demonstrate that BDNF administration increases resistance to fatigue independent of exercise status in a treadmill test to exhaustion. This longer time to exhaustion with the BDNF‐treated mice was similar to what was observed in the endurance‐trained mice, indicating that BDNF plays a role in the physiological changes that occur in skeletal muscle in response to ET. Indeed, isolated muscle preparations demonstrated that BDNF enhances EDL's maximal rate of relaxation to a similar extent as exercise alone. In support of this finding, BDNF has been shown to recover contractile strength in fast‐twitch muscle fibers of mouse models of Kennedy's disease, suggesting a potential influence of BDNF on muscle contractile properties.^51^ Together these results provide evidence that BDNF enhances muscle function and resistance to fatigue to a similar extent as ET.

ET is well known to result in a higher mitochondrial protein content and improved mitochondrial function which can result in enhanced muscle function.^52^ Our ET protocol did elevate mitochondrial proteins (PDH and citrate synthase in the SOL and citrate synthase in the EDL), demonstrating the efficacy of the protocol in increasing markers of oxidative energy metabolism. These results are similar to a study conducted by Siu et al., who used a similar 8‐week progressive ET protocol, finding SOL citrate synthase content to be 25% higher 48‐h after the final training session.^53^ Ringholm et al. showed voluntary wheel running from 3 to 15 months of age increased the EDL PDH content of wild‐type mice fed a chow diet compared with untrained mice.^54^ Here we show that BDNF treatment alone increased COXIV and citrate synthase in the EDL but did not alter mitochondrial protein content in the SOL, perhaps because the mitochondrial content in the SOL is already elevated. These results indicate that BDNF may play a role in increasing mitochondrial proteins with exercise however, the contribution that BDNF supplementation has on mitochondrial protein expression differs slightly from that of ET.

It has previously been demonstrated that BDNF can play a role in rodent food intake,^55^ although the doses of BDNF utilized in these previous studies were supraphysiological (~20 mg/kg/day) compared with the more physiological dose utilized in our study (0.5 mg/kg bw). Given this previous work, it was important to track body mass and food intake in our study. We demonstrate that BDNF treatment resulted in a lower body mass and food intake compared with the control and exercise groups. These findings are consistent with previous literature from Nakagawa et al.^55^ and Ono et al.^56^ who showed that oral supplementation or subcutaneous injection of ~3 μL and 1–70 mg/kg respectively of BDNF to significantly reduce food intake through appetite suppression in a *db/db* obese mice model.^55^, ^56^ To further support the role for BDNF in appetite regulation, BDNF has been implicated in leptin regulation, a peptide hormone that plays an important role in energy balance and inhibition of hunger. Dysregulation through compromised BDNF signaling within the hypothalamic neural circuits, due to a lack of leptin‐induced BDNF mRNA translation can lead to leptin resistance, resulting in obesity.^57^ Given these findings, one could speculate that an abundance of BDNF could result in hyperactivity of the hypothalamus, resulting in an anorexigenic effect of BDNF. While more work is required to fully interrogate the effects of BDNF on appetite regulation and body mass, our work here demonstrates that chronic BDNF treatment results in reduced body mass and food intake, suggesting a potential target for weight management while maintaining muscle health.

This work contributes to our knowledge of skeletal muscle BDNF content and the responsiveness to acute and chronic exercise and provides compelling evidence for a role of BDNF in skeletal muscle adaptations to ET. Our findings demonstrate that proBDNF content is higher in oxidative muscles and increases with acute exercise, suggesting its importance in exercise‐induced adaptations. The similar effect of BDNF on ET on the treadmill test to exhaustion highlights a role for BDNF in enhancing endurance performance. Differential effects of BDNF on contractile properties and mitochondrial markers in glycolytic (EDL) versus oxidative (SOL) muscles underscore the complexity of BDNF's action in skeletal muscle. Considering the higher levels of BDNF and mitochondrial markers naturally found in SOL muscles from sedentary mice, it is possible that the oxidative SOL muscle may be less responsive to additional BDNF treatment compared with the EDL, which has relatively lower BDNF and mitochondrial content. While our results strongly suggest that BDNF contributes to the skeletal muscle adaptations observed with ET, further investigations are necessary to elucidate the precise mechanisms and determine if BDNF is indispensable for these adaptations.

## AUTHOR CONTRIBUTIONS

Alexander D. Brown, Brian D. Roy, Val A. Fajardo, and Rebecca E. K. MacPherson designed research. Alexander D. Brown, Alexander D. Marko, Daniel M. Marko, Bradley J. Baranowski, Sebastian Silvera, Michael S. Finch, Alex J. Yang, Roopan Dhaliwal, and Chantal R. Ryan performed research. Alexander D. Brown, Alexander D. Marko, Val A. Fajardo, and Rebecca E. K. MacPherson analyzed data. Alexander D. Brown and Rebecca E. K. MacPherson wrote the paper. Everyone reviewed and edited the paper.

## DISCLOSURES

The authors declare that the research was conducted in the absence of any commercial or financial relationships that could be construed as a potential conflict of interest.

## Data Availability

The data that support the findings of this study are available on request from the corresponding author.
